# *Litsea japonica* Extract Inhibits Aldose Reductase Activity and Hyperglycemia-Induced Lenticular Sorbitol Accumulation in db/db Mice

**DOI:** 10.1155/2015/747830

**Published:** 2015-02-23

**Authors:** Junghyun Kim, Chan-Sik Kim, Eunjin Sohn, Yun Mi Lee, Kyuhyung Jo, Jin Sook Kim

**Affiliations:** Korean Medicine Based Herbal Drug Development Group, Herbal Medicine Research Division, Korea Institute of Oriental Medicine, Daejeon 305-811, Republic of Korea

## Abstract

Aldose reductase (AR) is the first and rate-limiting enzyme of the polyol pathway. AR-dependent synthesis of excess polyols leads to lens opacification in diabetic cataract. The purpose of this study is to investigate the protective effect of *Litsea japonica* extract (LJE) on diabetes-induced lens opacification and its protective mechanism in db/db mice. Seven-week-old male db/db mice were treated with LJE (100 and 250 mg/kg body weight) once a day orally for 12 weeks. LJE dose dependently inhibited rat lens aldose reductase activity *in vitro* (IC_50_ = 13.53 ± 0.74 *µ*g/mL). In db/db mice, lens was slightly opacified, and lens fiber cells were swollen and ruptured. In addition, lenticular sorbitol accumulation was increased in db/db mice. However, the administration of LJE inhibited these lenticular sorbitol accumulation and lens architectural changes in db/db mice. Our results suggest that LJE might be beneficial for the treatment of diabetes-induced lens opacification. The ability of LJE to suppress lenticular sorbitol accumulation may be mediated by the inhibition of AR activity.

## 1. Introduction

Cataract is a major cause of visual impairment in patients with diabetes. The pathogenesis of diabetic cataract is well known to involve some biochemical pathways, such as advanced glycation end products, polyol pathway, and oxidative stress [1]. Hyperglycemia in diabetic patients is known to elicit the activation of aldose reductase (AR) [2]. AR is the first and rate-limiting enzyme of the polyol pathway [3]. Polyols, such as sorbitol, physiologically accumulate inside the cells. Under diabetic conditions, AR leads to a conversion of excess glucose to sorbitol in tissues [4]. The accumulation of sorbitol in lens fiber cells leads to an increase of lens osmotic stress [5]. AR-dependent synthesis of excess polyols has been implicated as one of the mechanisms leading to diabetic cataracts [6]. Thus, inhibition of lens AR pathway could be one of the strategies to prevent diabetic cataract.

Some herbal extracts have the inhibitory effect on AR activity [7].* Litsea japonica* (Thunb.) Jussieu is a Korean native plant and has been utilized as a vegetable food [8]. In our previous reports, the extract of* L. japonica* prevented the development of diabetic nephropathy in diabetic mice [9] and ameliorated diabetes-induced retinal vascular leakage [10]. Although various preventive effects of* L. japonica* extract on diabetes-related complications have been reported, the effect on diabetic cataracts is still unknown. The pharmacological effect of* L. japonica* as an AR inhibitor is not yet well defined. Therefore, in this study, we examined the inhibitory effect of an ethanol extract of* L. japonica* on AR activity. We also evaluated the prevented effect of the extract of* L. japonica* on diabetes-induced lenticular sorbitol accumulation in db/db mouse, an animal model of type 2 diabetes.

## 2. Materials and Methods

### 2.1. Preparation of* L. japonica* Extract (LJE)

The aerial parts of* L. japonica* were obtained from the Jeju province in South Korea. A voucher specimen was deposited in the Herbarium of Korea Institute of Oriental Medicine, South Korea. The dried and ground plant material (3 kg) was extracted with EtOH (3 × 20 L) by maceration at room temperature for 3 days. The extracts were combined and concentrated* in vacuo* at 40°C to give an EtOH extract (390 g). The HPLC fingerprint and contents of major compounds of LJE are described in the previous report [10].

### 2.2. *In Vitro* Aldose Reductase Assay

To determine the inhibitory activity of LJE on AR, LJE and 3,3-tetramethyleneglutaric acid, a well-known AR inhibitor, were assayed according to previously described methods [11]. Briefly, the mixture solution contained 135 mM Na, K-phosphate buffer (pH 7.0), 100 mM Li_2_SO_4_, 0.03 mM NADPH, 0.04 mM D,L-glyceraldehyde, and 100 *μ*L of a purified rat lens AR with or without 50 *μ*L of LJE or positive inhibitor, in a total volume of 1.0 mL. The reaction was initiated by adding NADPH at 37°C and stopped by adding 0.3 mL of 0.5 N HCl. Then 1 mL of 6 N NaOH containing 10 mM imidazole was added, and the mixture was incubated at 60°C for 10 min to convert NADP to a fluorescent product. The fluorescence was measured with a spectrofluorophotometer (Ex/Em = 360 nm/460 nm; Synergy HT, Bio-Tek, VT, USA). We calculated the 50% inhibition concentration (IC_50_) of AR activity.

### 2.3. Animals and Experimental Design

Male C57BL/KsJ db/db mice (db/db) and their lean littermates (db/+) were obtained from Japan SLC (Shizuoka, Japan). At 8 weeks of age, mice were randomly divided into four groups (*n* = 10). LJE was dissolved in 0.5% w/v carboxyl methylcellulose solution. Two groups of db/db mice received daily gastric gavage of LJE at 100 and 250 mg/kg, respectively, for 12 weeks. One group of db/db mice and normoglycemic group of db/+ mice were given the same amount of vehicle gavage. The blood glucose level was monitored consecutively. Mice were examined weekly in a dark room using an ophthalmoscope. At necropsy, eyes were enucleated under deep anesthesia following an intraperitoneal injection of pentobarbital sodium (30 mg/kg body weight). The lenses were excised from the eyeball and then photographed under an optical microscope. All procedures involving rats were approved by the Korea Institute of Oriental Medicine Institutional Animal Care and Use Committee.

### 2.4. Sorbitol Assay

A 10% lens homogenate was prepared in a 50 mM phosphate buffer, pH 7.4. Sorbitol levels in the lens were measured using an enzymatic method [12]. Briefly, a total of 1 mL of extract from each lens was mixed with 2 mL glycine buffer (0.05 M, pH 9.4) containing 2 mM NAD+ and 0.05 mL sorbitol dehydrogenase (25.6 U/mL) and incubated at room temperature for 60 min. After incubation, fluorescence of the generated NADH was measured at an excitation wavelength of 366 nm and emission wavelength of 452 nm using a spectrofluorometer (Synergy HT, Bio-Tek, VT, USA). Sorbitol levels in each extract were calculated from a calibration curve of D-sorbitol. Sorbitol content in the lens was expressed as *μ*mol/g wet weight.

### 2.5. Analysis of Lens Fiber Alteration

At necropsy, the enucleated eyes were fixed in 10% neutralized formalin for 24 h and embedded in paraffin. To analyze lens fiber alteration, fiber cells were visualized by labeling their membranes with wheat germ agglutinin. The lens sections were incubated with 2.5 mg/mL rhodamine-conjugated wheat germ agglutinin (Vector Laboratories, CA, USA) for 60 minutes. The sections were examined with a fluorescence microscope (BX51, Olympus, Tokyo, Japan).

### 2.6. Statistical Analysis

Statistical evaluation of the results was performed using Student's *t*-test and a one-way analysis of variance (ANOVA) followed by Tukey's multiple comparison test using GraphPad Prism 4.0 software (Graph pad, CA, USA).

## 3. Results

### 3.1. LJE Inhibits Rat Lens AR Activity* In Vitro*

LJE was tested for its inhibitory activity on rat lens AR. As shown in Table 1, LJE dose dependently inhibited AR activity (IC_50_ = 13.53 ± 0.74 *μ*g/mL).

### 3.2. Blood Glucose Levels

As shown in Table 2, the blood glucose was significantly increased in* db/db* mice (*P* < 0.01 versus normal). The administration of LJE at dose of 250 mg/kg slightly decreased blood glucose, but this difference was not statistically significant.

### 3.3. Lens Opacification and Lens Fiber Alteration

**Figure 1:**
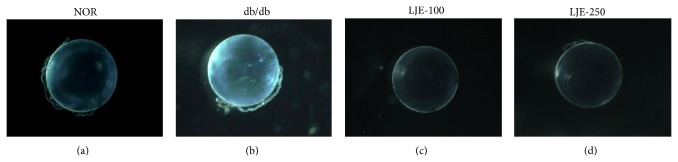
Lens opacity. Representative image of lenses in each group. NOR, normal db/+ mice; db/db, diabetic* db/db* mice; LJE-100, db/db mice treated with LJE (100 mg/kg); LJE-250, db/db mice treated with LJE (250 mg/kg).

**Figure 2:**
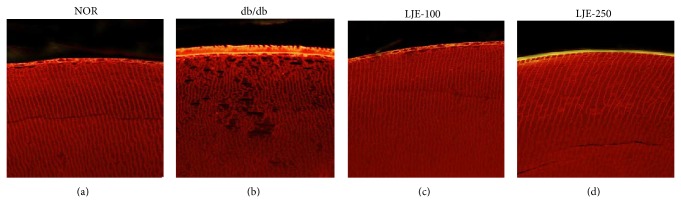
Lens fiber alteration. Lens sections were labeled with rhodamine-conjugated wheat germ agglutinin. Fiber cell swelling and membrane rupture were observed in db/db mice.

At the end of the twelve week study, no lenses displayed cataract formation in normal db/+ mice. The lenses of vehicle-treated db/db mice became slight opaque. However, none of LJE-treated db/db mice developed lens opacity (Figure 1). Microscopically, the lenses of normal db/+ mice consistently showed a perfect crystalline packing of fiber cells. In db/db mice, lens fiber cell swelling and multiple membrane rupture were detected. However, this alteration of lens fiber cells in db/db mice was prevented by LJE treatment (Figure 2).

### 3.4. Lenticular Sorbitol Accumulation

**Figure 3:**
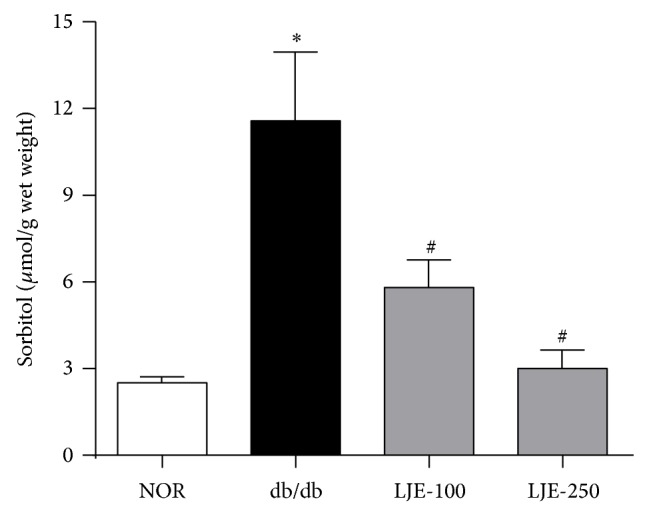
Lenticular sorbitol levels. Values in the bar graphs represent means ± SEM, *n* = 8. ^*^ *P* < 0.01 versus normal db/+ mice, ^#^ *P* < 0.01 versus vehicle-treated db/db mice.

There was an increase of lenticular sorbitol levels in vehicle-treated db/db mice when compared to normal db/+ mice. However, oral administration of LJE dose dependently reduced diabetes-induced lenticular sorbitol accumulation (Figure 3).

## 4. Discussion

Some herbal extracts have showed the potential anti-cataract activities in diabetic animals [13, 14]. In the present study, LJE has an inhibitory activity on AR* in vitro*. In db/db mice, LJE prevented lens opacification and lens fiber alteration. In addition, lenticular sorbitol accumulation was significantly reduced by LJE administration in db/db mice. Although,* L. japonica* has been utilized as a vegetable food [8], its safety profile has not been adequately documented. However, in our previous and present studies, LJE has been shown not to cause any biochemical and histopathological changes in mice [9, 10].

AR is a key enzyme in the polyol pathway and plays an important role in cataractogenesis in diabetic conditions [15]. Diabetes-induced lens opacification is closely related to AR-catalyzed production of sugar alcohols [16, 17]. At this time, there are no commercially available anti-AR drugs for diabetic cataract. The* in vivo* effect of AR inhibitors such as tolrestat [18], epalrestat [19], SNK-860 [20], and SG-210 [21] on cataractogenesis is inadequate in spite of their potent ameliorating effect on AR* in vitro*. LJE showed the potent anti-AR activity* in vitro*. Moreover, the lenticular sorbitol accumulation osmotically draws water into the lens fiber cells, causing lens fiber swelling and rupture in db/db mice. The administration of LJE suppressed these lenticular sorbitol accumulation and lens architectural changes in db/db mice. In the lens, osmotic stress induced by sorbitol accumulation has been considered a major causative factor in the pathogenesis of diabetic cataracts [22]. Our results suggest that LJE can prevent diabetes-induced lens opacification through an inhibition of AR activity as well as sorbitol accumulation in lens.

Herbal extracts are known to have various advantages of synergy and interactions among the various phytochemicals. Three flavonoids, epicatechin, quercitrin, and afzelin, were identified in LJE [10]. Epicatechin, quercitrin, and afzelin are the potent AR inhibitors, with IC_50_ values of 79 *μ*M, 0.17 *μ*M, and 5.54 *μ*M, respectively [23–25]. Moreover, catechin prevented* N*-methyl-*N*-nitrosourea-induced cataracts and lens epithelial cell apoptosis in rat [26]. Therefore, the inhibitory effect of LJE on cataractogenesis may be a result from synergistic interactions of these compounds.

In summary, LJE suppressed AR activity* in vitro*. In addition, the administration of LJE prevented lens opacification and lenticular sorbitol accumulation in db/db mice. Although the pathogenesis of sugar cataract is multifactorial, polyol pathway is a major pathway for the progression of diabetic cataract. It could be reasonable to assume that the preventive effect of LJE on diabetic cataract might be at least partly attributed to its influence on AR activity. Thus, LJE could be beneficial agent by protecting against diabetes-induced lens opacification.

## Figures and Tables

**Table 1:** Inhibition of rat lens aldose reductase by LJE.

	Concentration (*μ*g/mL)	Inhibition (%)	IC_50_ value^a^ (*μ*g/mL)
LJE	10	42.38 ± 3.58	13.53 ± 0.74
	12.5	47.35 ± 1.72	
	15	53.64 ± 4.14	

3,3-Tetramethyleneglutaric acid	3.7	31.42 ± 5.71	5.34 ± 0.89
	5.5	56.42 ± 9.60	
	7.4	69.69 ± 8.15	

^a^The inhibitory effect is expressed as the mean of four experiments. IC_50_ values were calculated from dose inhibition curves.

**Table 2:** Blood glucose levels.

	NOR	db/db	LJE-100	LJE-250
Blood glucose (mmol/L)	6.7 ± 2.0	43.0 ± 0.8^*^	38.6 ± 9.8	32.5 ± 12.0

NOR, normal db/+ mice; db/db, diabetic *db/db* mice; LJE-100, db/db mice treated with LJE (100 mg/kg); LJE-250, db/db mice treated with LJE (250 mg/kg). All data are expressed as the mean ± SEM. ^*^ *P* < 0.01 versus the NOR group.
